# Deep‐MSIM: Fast Image Reconstruction with Deep Learning in Multifocal Structured Illumination Microscopy

**DOI:** 10.1002/advs.202300947

**Published:** 2023-07-09

**Authors:** Jianhui Liao, Chenshuang Zhang, Xiangcong Xu, Liangliang Zhou, Bin Yu, Danying Lin, Jia Li, Junle Qu

**Affiliations:** ^1^ State Key Laboratory of Radio Frequency Heterogeneous Integration Key Laboratory of Optoelectronic Devices and Systems of Ministry of Education and Guangdong Province College of Physics and Optoelectronic Engineering Shenzhen University Shenzhen 518060 China

**Keywords:** deep learning, image reconstruction, multifocal structured illumination microscopy, U‐net

## Abstract

Fast and precise reconstruction algorithm is desired for for multifocal structured illumination microscopy (MSIM) to obtain the super‐resolution image. This work proposes a deep convolutional neural network (CNN) to learn a direct mapping from raw MSIM images to super‐resolution image, which takes advantage of the computational advances of deep learning to accelerate the reconstruction. The method is validated on diverse biological structures and in vivo imaging of zebrafish at a depth of 100 µm. The results show that high‐quality, super‐resolution images can be reconstructed in one‐third of the runtime consumed by conventional MSIM method, without compromising spatial resolution. Last but not least, a fourfold reduction in the number of raw images required for reconstruction is achieved by using the same network architecture, yet with different training data.

## Graphical abstract

This work develops a deep‐learning‐based algorithm which reconstructs the super‐resolution image directly from raw images of multifocal structured illumination microscopy. The method is validated on microtubules, static and live‐cell mitochondria, and live‐zebrafish at a depth of 100 µm, and achieves state‐of‐the‐art performance with one‐fourths numbers of raw images and one‐third of the runtime required by conventional reconstruction method.

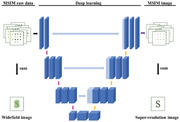

## 1 Introduction

In the past two decades, the impressive developments of super‐resolution fluorescence microscopy allow the researchers to observe and investigate biological structures and life fundamental processes at a lateral resolution of 20 nm.^[^ ^1^, ^2^ ^]^ Compared with other super‐resolution techniques, such as stochastic optical reconstruction microscopy (STORM)^[^ ^3^ ^]^ and stimulated emission depletion (STED) microscopy,^[^ ^4^, ^5^ ^]^ the structured illumination microscopy (SIM)^[^ ^6^, ^7^ ^]^ provides a modest lateral resolution. Nevertheless, SIM needs far fewer raw images than that in STORM to reconstruct a super‐resolution image, and it needs far lower illumination intensity than that in STED microscopy. Linear SIM is also compatible with the most common fluorescent dyes, unlike STORM or STED. Consequently, SIM is particularly suitable for long‐term live‐cell imaging.

Despite these advances, SIM encounters challenges when imaging thick samples or imaging at a greater depth. Multifocal SIM (MSIM) is therefore proposed to extend the depth penetration of SIM, which uses a sparse focus array of excitation and replaces the single detector with a detector array.^[^ ^8^, ^9^ ^]^ Additionally, MSIM can dramatically increase the imaging speed via the parallelization of the image scanning microscopy (ISM). There are four important steps in the reconstruction procedure of MSIM, including pinholing, local scaling, summing, and deconvolution, and the final image can achieve a twofold spatial resolution better than the diffraction limit and axial resolution of ≈400 nm.^[^ ^10^ ^]^ Thus, efficient algorithms for image reconstruction are crucial for MSIM.

A variety of algorithms have been presented to process MSIM data. For instance, Ströhl et al. develop a joint Richardson–Lucy deconvolution algorithm to reconstruct MSIM raw images with enhanced resolution and good quality.^[^ ^11^ ^]^ Wu et al. achieve a higher‐than‐twofold resolution enhancement with a sparse Bayesian learning algorithm.^[^ ^12^ ^]^ In Feng et al. method, nine raw multifocal images with enhanced modulation depth are first produced, and then the reconstructed images are summed together to obtain the final super‐resolution image with good contrast.^[^ ^13^ ^]^ Zhang et al. propose an enhanced optical sectioning super‐resolution method that can improve optical sectioning capability and the lateral resolution by combining the ISM with the standard deviation method.^[^ ^14^, ^15^ ^]^ However, the above approaches aim to enhance the resolution and may be at the expense of speed. Many raw images and iteration times are required for the reconstruction; besides, four steps in MSIM are separately accomplished in the reconstruction procedures of these methods. This is complicated and time‐consuming, limiting extensive adoption of MSIM for practical use.

To address the issues, we propose a fast reconstruction algorithm based on deep learning, which achieves direct end‐to‐end image transformation rather than implementing, respectively, and distinguishing the four main steps in MSIM. Deep learning has more complex ways of connecting layers and larger amount of computing power in training than previous networks, and another remarkable advantage is automatic feature extraction that deep learning has over conventional machine learning algorithms.^[^ ^16^, ^17^ ^]^ More importantly, it is different from classical optimization approaches that need explicit analytical modeling and prior knowledge.^[^ ^18^ ^]^ Recent applications of deep learning to image processing have been implemented successfully in a variety of research fields.^[^ ^19^, ^20^, ^21^, ^22^, ^23^ ^]^ Super‐resolution fluorescence microscopy has recently benefited from deep learning to improve resolution or to reconstruct high‐resolution images, and these studies demonstrate infinite possibilities of deep learning in super‐resolution microscopy.^[^ ^24^ ^]^ Yao et al. focus on developing a reconstruction algorithm suited for high‐density molecule localization and live‐cell STORM imaging.^[^ ^25^ ^]^ Speiser et al. develop deep context dependent that is able to localize single emitters at high density in three dimensions with highest accuracy for a large range of imaging modalities and conditions.^[^ ^26^ ^]^ Wang et al. present a deep‐learning‐based framework to achieve super‐resolution and cross‐modality image transformations in fluorescence microscopy.^[^ ^27^ ^]^ Huang et al. transforms diffraction‐limited confocal microscopy images to match the resolution acquired with STED microscope, showing potential that the deep learning method is able to infer super‐resolved live‐cell images by training the model with only the static images.^[^ ^28^ ^]^ Jin et al. develop an efficient deep learning assisted SIM (DL‐SIM) reconstruction algorithm that needs fewer frames of raw SIM images and works under low light conditions, thus increasing acquisition speed and reducing photobleaching.^[^ ^29^ ^]^ Qiao et al. present a deep Fourier channel attention network and its derivative trained with generative adversarial network (GAN), which achieves robust reconstruction of SIM images under low signal‐to‐noise (SNR) conditions.^[^ ^30^ ^]^ Li et al. achieve ≈120 nm isotropic resolution for 3D SIM based on a deep learning method that can be combined with denoising to facilitate volumetric imaging spanning dozens of timepoints.^[^ ^31^ ^]^ Zhang et al. demonstrate an improved generative adversarial network for ISM reconstruction that can be trained by simulation data and has good generalization.^[^ ^32^ ^]^ To the best of our knowledge, none of other works so far has proposed and demonstrated the capability of deep learning to reconstruct directly from raw MSIM images.

This work focuses on developing a fast MSIM image reconstruction algorithm by leveraging deep learning, and we term it Deep‐MSIM. The proposed scheme is very fast, and produces images with quality comparable or better than conventional MSIM reconstruction method. We demonstrate its superior algorithmic performance on diverse biological samples. The model, trained with only the simulated data, can be used to achieve in vivo imaging at a depth of 100 µm, capturing the live zebrafish with fine structures and high resolution under scattered and out‐of‐focus light conditions. More importantly, we further increase the speed of MSIM imaging by reducing the number of raw images required for reconstruction by fourfold. This is fulfilled by devising the training data pairs elaborately.

## 2 Results

### 2.1 Simulated Data Analysis

**Figure 1:**
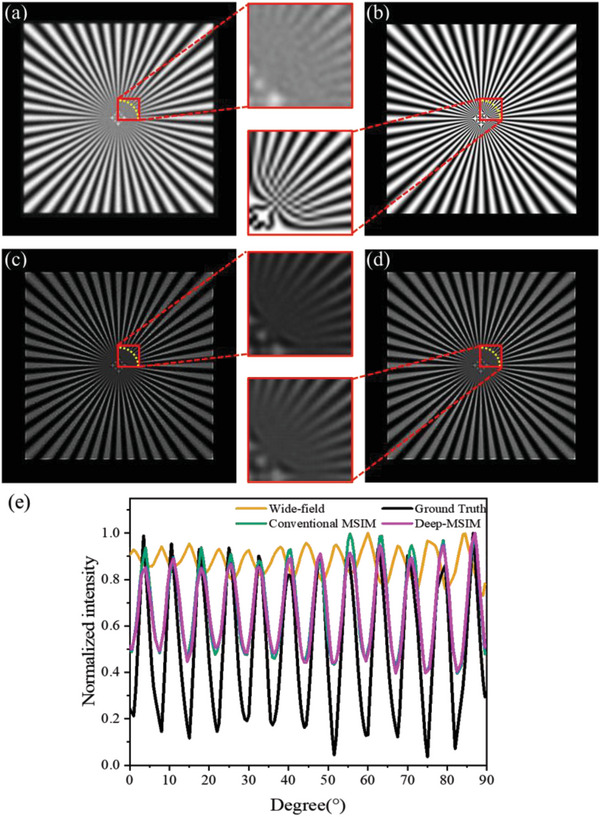
Comparison of conventional MSIM and Deep‐MSIM on simulated data. a) Sum of 256 frames of raw images. b) Ground truth image. (c) and (d) are reconstructed images respectively using conventional MSIM and Deep‐MSIM. Red squares in the middle are correspondingly magnified views. e) Plots of intensity profiles along the yellow dotted arcs.

We validate the performance of the proposed algorithm using both simulated and real MSIM. A Gaussian model point spread function (PSF) with the emission light λ_em_ = 560nm and numerical aperture (NA) NA = 1.1 is adopted. The image pixel size is set to 130 nm according to the microscope system employed in our experiments. Poisson noise is introduced to evaluate performance under nonideal condition. 256 frames of raw MSIM images are simulated and they are added to form a wide‐field image in **Figure** **1**a. Figure 1b–d displays the ground truth, as well as the resulting super‐resolution images using conventional reconstruction method and the proposed Deep‐MSIM method, respectively. We can observe that conventional MSIM and our Deep‐MSIM produce similar quality results. This is also consistent with the intensity profiles along the yellow dotted arcs in Figure 1e. However, the super‐resolution image is obtained in 6.42 s by using our method, one order of magnitude shorter than 76.13 s of conventional MSIM.

To achieve the best reconstruction results, the noise level for the training data should in principle be precisely matched to that of the experimental data. However, it is difficult to measure the accurate noise level, and it will be time consuming if we train the network for each different noise level. In our work, after evaluating an approximate noise level of the experimental data, we select a relative large value within the noise range for the generation of simulated training examples. The network trained with high SNR data, data with multiple SNR values and low SNR data are, respectively, tested on raw images with different noise levels, and the results are presented in Figure S1, Supporting Information. Larger peak signal‐to‐noise ratio (PSNR) values in Figure S1g,h,i (Supporting Information) explicitly indicate that the capacity of the network for reconstructing raw images with high and low noise levels are both improved. This also enables the network to be trained only one time, and the resulting weights and the trained network are appropriate to different noise levels.

### 2.2 Real Data Analysis

**Figure 2:**
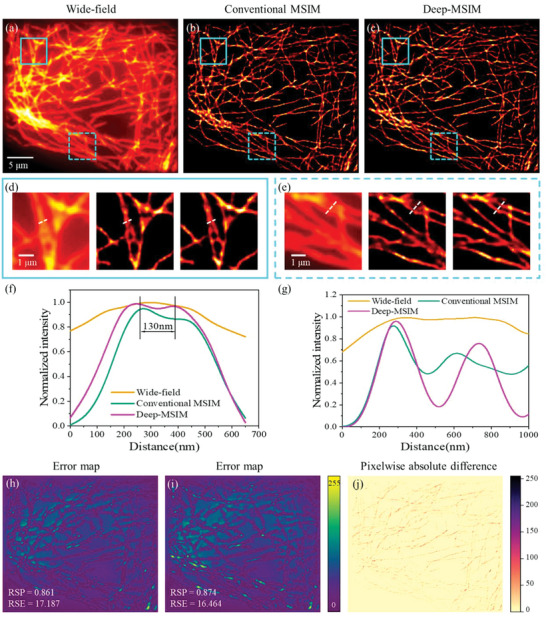
Comparison of conventional MSIM and Deep‐MSIM on experimental data of microtubules. a) Wide‐field image summed from 1058 frames of raw MSIM images. (b) and (c) are reconstructed images respectively using conventional MSIM and Deep‐MSIM. (d) and (e) are correspondingly magnified views of cyan solid and dotted squares. (f) and (g) are plots of intensity profiles along the white dotted lines. (h) and (i) are error maps between the network input wide‐field image and conventional MSIM reconstructed image, or Deep‐MSIM output image, respectively. (j) is the pixelwise absolute difference image between (b) and (c).

Next, we verify the practicability of the algorithm by applying it to reconstruct real MSIM data. The imaging of thin BSC‐1 cells stained with CdSe/ZnS is performed on custom built two‐photon MSIM system (Experimental Section). Compared to the wide‐field image in **Figure** **2**a, significant resolution enhancement is achieved in both the reconstructed images using the conventional MSIM method (Figure 2b) and the presented Deep‐MSIM method (Figure 2c). These two methods reconstruct more obvious structures, and two microtubules unresolved and incorrectly merged in the wide‐field image are clearly separated, as exemplified by the magnified views. The intensity profiles in Figure 2f show that Deep‐MSIM can resolve two close microtubules with a distance of 130 nm. Deep‐MSIM not only yields image reconstruction results that are comparable to or slightly better than traditional algorithm but also reduces the runtime from 101.80 to 37.03 s.

A common concern for deep‐learning‐based algorithms is the possible spatial artifacts emerged in the reconstructed image. We quantify the errors for both methods using the Fiji^[^ ^33^ ^]^ software plugin (NanoJ‐SQUIRREL).^[^ ^34^ ^]^ The SQUIRREL plugin also provides two globally averaged scores: resolution scaled error (RSE) and resolution scaled Pearson coefficient (RSP). The former calculates the root‐mean‐square error between the reference and resolution‐scaled image and is more sensitive to differences in brightness and contrast, while the later helps to assess the image qualities across modalities by quantifying their Pearson correlation coefficients (Experimental Section).^[^ ^27^ ^]^ In our implementation, the “Reference image” is set to the input raw MSIM image, and the “Super‐resolution reconstruction” is set to the conventional MSIM reconstructed image or Deep‐MSIM output image. The results in Figure 2h,i reveal that Deep‐MSIM output image does not generate noticeable super‐resolution related artifacts and in fact has lower level of spatial mismatch error than that the conventional MSIM reconstructed image has.

To directly visualize the mismatch between our network output image and conventional MSIM reconstructed image, we compute the pixelwise absolute difference between the results from two methods. Figure 2j displays the residual image where the remains are mainly caused by the different pixelwise intensities in Figure 2b,c, yet the overall morphology of the reconstructed microtubules structures are similar.

**Figure 3:**
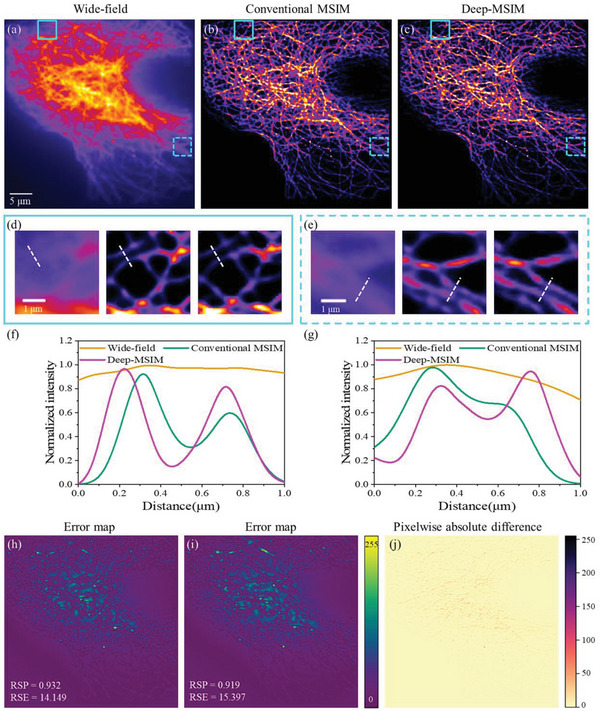
Comparison of conventional MSIM and Deep‐MSIM on experimental data of dense microtubules. a) Wide‐field image summed from 1764 frames of raw MSIM images. (b) and (c) are reconstructed images respectively using conventional MSIM and Deep‐MSIM. (d) and (e) are correspondingly magnified views of cyan solid and dotted squares. (f) and (g) are plots of intensity profiles along the white dotted lines. (h) and (i) are error maps between the network input wide‐field image and conventional MSIM reconstructed image, or Deep‐MSIM output image, respectively. (j) is the pixelwise absolute difference image between (b) and (c).

Another experiment is conducted on more densely distributed microtubules. The corresponding results are given in **Figure** **3** with the same layout as Figure 2. It is clear that the structure details are not discernable in the raw MSIM images, while both conventional MSIM and our method are capable of reducing blur and resolving finer details, as presented in Figure 3a–c. Deep‐MSIM promotes the reconstruction of the information, and the signal consequently becomes more pronounced over the noise. The magnified views and the intensity profiles in Figure 3d–g reveal that the reconstructed super‐resolution image by applying Deep‐MSIM has slightly higher image contrast than that from conventional reconstruction method, which means the effective rejection of out‐of‐focus light. Total super‐resolution image‐acquisition time of Deep‐MSIM and conventional method are 59.54 and 158.70 s, respectively. The comparison of error maps in Figure 3h,i, as well as the pixelwise absolute difference image in Figure 3j confirm the conclusion that Deep‐MSIM reconstruction (Figure 3c) is in good agreement with the image obtained from the traditional method (Figure 3b).

**Figure 4:**
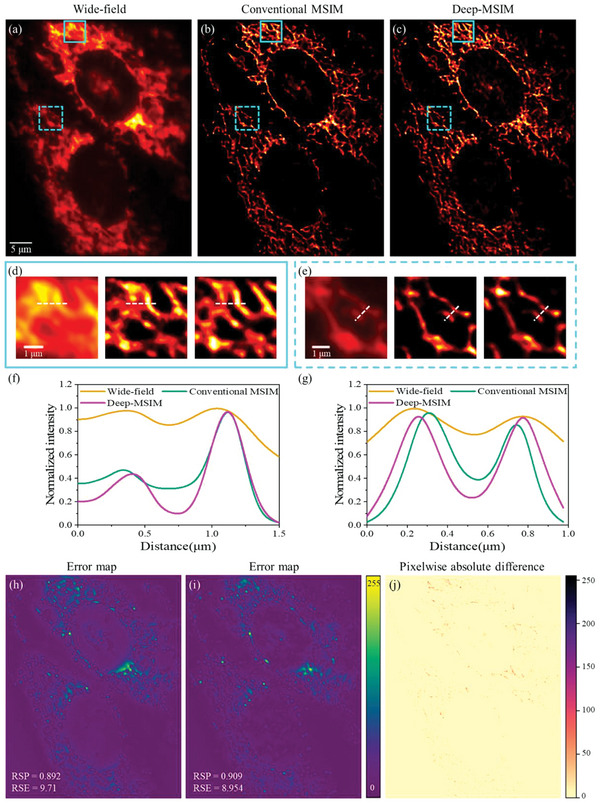
Comparison of conventional MSIM and Deep‐MSIM on experimental data of mitochondria. a) Wide‐field image summed from 1600 frames of raw MSIM images. (b) and (c) are reconstructed images respectively using conventional MSIM and Deep‐MSIM. d) and e) are correspondingly magnified views of cyan solid and dotted squares. (f) and (g) are plots of intensity profiles along the white dotted lines. (h) and (i) are error maps between the network input wide‐field image and conventional MSIM reconstructed image, or Deep‐MSIM output image, respectively. (j) is the pixelwise absolute difference image between (b) and (c).

To demonstrate the generalization of Deep‐MSIM, we apply the present framework to mitochondria, a very different biological structure, and another popular target of super‐resolution imaging studies.^[^ ^35^ ^]^ Previously trained model can be directly and successfully applied to the new structure without retraining, thereby highlighting the robustness of our method to biologically relevant structural variations. Similar improvement in image quality and resolution can be observed in **Figure** **4**, especially that the regions of dense and complex structures are better resolved and appear sharper. We measure the full width at half maximum (FWHM) of the intensity profiles in Figure 4g. Deep‐MSIM even has a slightly better intensity profile than that of conventional MSIM reconstruction method, with a FWHM of 247.8 versus 256.3 nm for the first peak, and the attained FWHM of the other peak with two methods are 192.4 and 173.2 nm, respectively. Again, the error maps in Figure 4h,i and few residual in Figure 4j demonstrate that no obvious feature mismatch between the network output image (Figure 4c) and conventional MSIM reconstructed image (Figure 4b), and Deep‐MSIM even obtains a lower RSE and a higher RSP, and better image contrast along the white lines in Figure 4d,e. This can be regarded as further evidence that the image quality is indeed improved and the runtime is indeed reduced by our proposed Deep‐MSIM. Deep‐MSIM also exhibits superior runtime, equivalent to reconstructing 30 frames of raw MSIM image per second, compared to 9 frames per second by conventional MSIM method. The rationale behind this result is that Deep‐MSIM has a better computational removal of out‐of‐focus light, and learns precise representation of the spatial structures.

### 2.3 Comparison with Representative Deep‐Learning‐Based Super‐Resolution Models

Considering the representativeness and performance of current deep‐learning‐based super‐resolution models, we select seven networks constructed with different architectures, including a recent development in deep Fourier channel attention network and its derivative trained with GAN (DFGAN);^[^ ^30^ ^]^ cross‐modality image transformation with U‐net^[^ ^36^ ^]^ architecture and the GAN mechanism (CrossmodalityGAN);^[^ ^27^ ^]^ photorealistic single image super‐resolution using a perceptual loss function in GAN architecture (SRGAN);^[^ ^37^ ^]^ a U‐net based approach for SIM super‐resolution image reconstruction with fewer input image and lower intensity (DL‐SIM);^[^ ^29^ ^]^ the pioneering work for STORM super‐resolution image reconstruction based on a fully convolutional encoder‐decoder network (Deep‐STORM);^[^ ^23^ ^]^ an enhanced deep super‐resolution network (EDSR) constructed with residual convolutional blocks in convolutional neural network ;^[^ ^38^ ^]^ and the earliest classical super‐resolution CNN (SRCNN) with a lightweight configuration.^[^ ^39^ ^]^

The sparse microtubules in Figure 2 and mitochondria in Figure 4 are employed as the input images, and Figures S2 and S3 (Supporting Information) show the super‐resolution images inferred from different models. Despite the fact that some of the aforementioned models have greatly advanced single‐image super‐resolution, all of them cannot produce acceptable results when each set of raw MSIM images is averaged out to a single frame of wide‐field image used as input. Instead, each frame of raw MSIM image is fed into the model to obtain the high‐resolution counterpart, and their summation is the desired super‐resolution image. The network outputs of SRGAN and SRCNN need postprocessing of enhancing contrast for better visualization.

To evaluate the performance of different super‐resolution models, we use three metrics, i.e., peak signal‐to‐noise ratio (PSNR),^[^ ^29^ ^]^ normalized root‐mean‐square error (NRMSE),^[^ ^30^ ^]^ and structural similarity index (SSIM),^[^ ^40^ ^]^ to quantitatively measure the quality of the super‐resolved images. PSNR and NRMSE numerically compute the pixel‐level data fidelity, and SSIM is very well matched to assess perceptual image quality (Experimental Section). With the conventional MSIM reconstruction served as ground‐truth, the statistical analysis in Tables S1 and S2 (Supporting Information) confirms that Deep‐MSIM significantly outperform other super‐resolution models in terms of all three metrics.

### 2.4 In Vivo Imaging

We then demonstrate the in vivo application of the proposed method by reconstructing MSIM images of the live zebrafish at a depth of 100 µm. Specifically, the imaging of live zebrafish (Tg(Xla.Eef1a1:mlsEGFP)) is performed on custom built two‐photon MSIM system, and 1764 frames of raw images are captured. We use the same network but retrain it by changing the training data. As in ref. [15], a normal PSF and a defocused PSF at the defocus distance of 3 µm, respectively, convolve with the training samples, and then the two convolution results are added to obtain the simulated training data. High Poisson noise level is also introduced for a reasonable simulation of low SNR condition.

**Figure 5:**
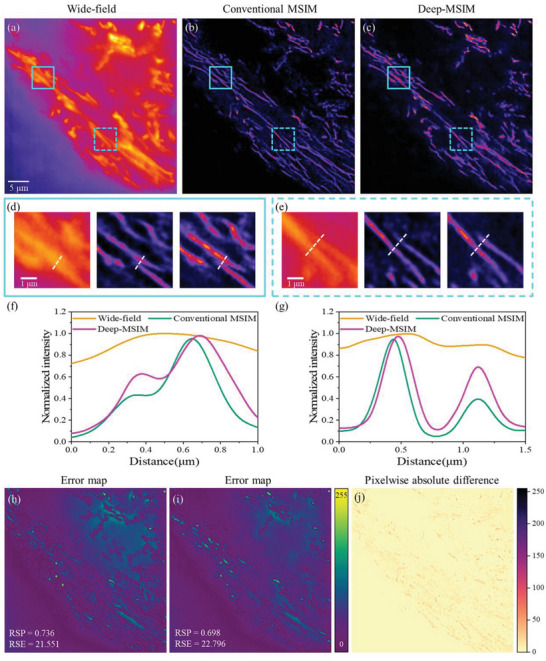
Comparison of conventional MSIM and Deep‐MSIM on experimental data of live zebrafish at a depth of 100 µm. a) Wide‐field image summed from 1600 frames of raw MSIM images. (b) and (c) are reconstructed images, respectively, using conventional MSIM and Deep‐MSIM. (d) and (e) are correspondingly magnified views of cyan solid and dotted squares. (f) and (g) are plots of intensity profiles along the white dotted lines. (h) and (i) are error maps between the network input wide‐field image and conventional MSIM reconstructed image, or Deep‐MSIM output image, respectively. (j) is the pixelwise absolute difference image between (b) and (c).

Comparing images before (**Figure** **5**a) and after Deep‐MSIM reconstruction (Figure 5c) reveals obvious improvements in resolution and signal. The intensive background noise has been suppressed, and the improvements are sufficient to resolve smaller somites in live zebrafish in addition to the larger and brighter structures observed in raw images. Deep‐MSIM achieves the desired super‐resolution image in 72.87 s, in contrast to the conventional MSIM method that requires 162.63 s. It should be noted that Deep‐MSIM reduces the execution time without deterioration to performance, as illustrated in Figure 5f,g where larger intensity fluctuation indicates that finer structures are reconstructed at a depth of 100 µm. In Figure 5g, the measured FWHM of conventional reconstruction method are 237.1 and 240 nm, respectively, while the intensity profile of Deep‐MSIM is moderately wider, with a FWHM of 253.6 and 246 nm, respectively. Since both methods use the simulated training data that do not contain background ground‐truth, similar to that discussed,^[^ ^41^ ^]^ the larger error in the upper right background region of Figure 5h,i is due to the fact that two approaches can suppress the background signal.

**Figure 6:**
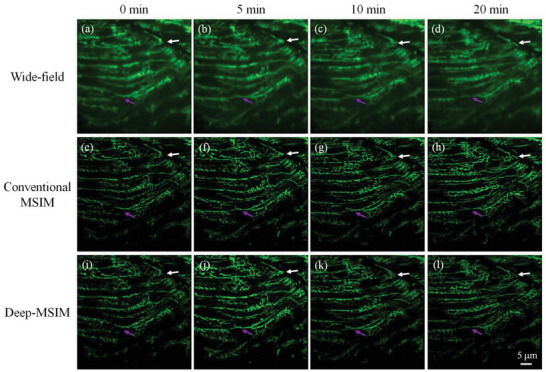
Comparison of conventional MSIM and Deep‐MSIM on experimental data of live zebrafish. (a–d) are time‐lapse wide‐field image summed from 1600 frames of raw MSIM images. (e–h) are time‐lapse reconstructed images using conventional MSIM. (i–l) are time‐lapse reconstructed images using Deep‐MSIM. The morphological changes are indicated by white and magenta arrows. Video S1 (Supporting Information) shows the same field of view to further highlight the success of Deep‐MSIM in revealing mitochondrial dynamics of the zebrafish.

We conduct two additional super‐resolution imaging experiments on live zebrafish and live Hela cell to observe the mitochondrial dynamics that is important for the maintenance of cellular functions.^[^ ^42^ ^]^ First, we reconstruct 1600×25 frames of raw MSIM images acquired from live zebrafish (Tg(Xla.Eef1a1:mlsEGFP)) with conventional method and Deep‐MSIM, and the results are shown in **Figure** **6**. The motions of the mitochondria are visualized clearly, for example, the long mitochondrion continues to migrate along the direction of adjacent circular mitochondria and gradually divides into two segments, and two close mitochondria fuse into a very slender one, as marked by white and magenta arrows, respectively. We reconstruct each frame of super‐resolution image in one‐third of the runtime consumed by conventional MSIM method, with 56.16 versus 231.3 s. Second, we record a long‐term (36 min) super‐resolution imaging of live‐cell mitochondria (Hela cell labeled with Mito‐tracker Green) with two methods. The mitochondrial fission process can be captured in the corresponding results (Figure S4, Supporting Information), indicated by the white and magenta arrows, the mitochondria are in a typical bubble structures at ≈8 min, and then they separate to form individual line structure at ≈35 min. We also notice that each super‐resolution image is reconstructed in 52.09 s by Deep‐MSIM using 1600 frames of raw images that are acquired at 60 s. This implies the potential of our method for real‐time MSIM super‐resolution image reconstruction. Videos S1 and S2 (Supporting Information) further highlight the success of Deep‐MSIM in revealing the subtle morphological changes and dynamics of the mitochondria in zebrafish and Hela cell.

### 2.5 Reconstruction with Fewer Images

**Figure 7:**
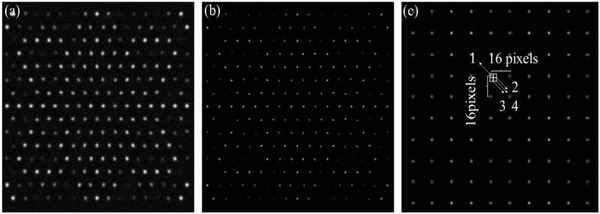
Generation of new training data pairs. a) A raw image produced under the multifocus array in (c). b) Summation of four reconstructed MSIM images. c) The multifocus array. The distance between the laser foci is set as 16 pixels in both horizontal and vertical direction.

In spite of the fact that Deep‐MSIM is much faster than conventional MSIM reconstruction algorithm, it does not reduce the amount of raw data required to reconstruct a super‐resolution image. Accordingly, Deep‐MSIM is expected to accelerate MSIM imaging by using fewer frames of raw images for reconstruction. We still use the same network and retrain it with newly generated low‐and‐high resolution image pairs. The low resolution image (**Figure** **7**a) is produced under the multifocus array in Figure 7c. Then we shift the laser foci in horizontal and vertical direction, such that the laser focus originally located at pixel 1 changes position to the neighboring pixels 2–4, as marked in Figure 7c. Four frames of raw images are therefore acquired. The summation of their correspondingly reconstructed MSIM images, rather than the reconstruction result of one single frame, is used as the high resolution image (Figure 7b). What's more, the step size in both horizontal and vertical direction is set as 2 during the collection process, resulting in a fourfold reduction in the number of required raw data.

**Figure 8:**
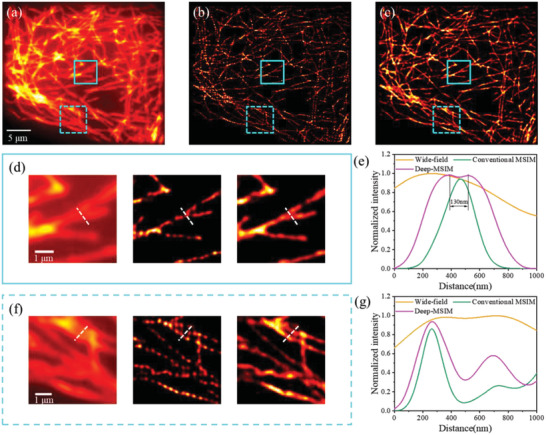
Comparison of conventional MSIM and Deep‐MSIM with fewer frames of raw experimental data. a) Wide‐field image summed from 274 frames of raw MSIM images. (b) and (c) are reconstructed images respectively using conventional MSIM and Deep‐MSIM. (d) and (f) are correspondingly magnified views of cyan solid and dotted squares. (e) and (g) are plots of intensity profiles along the white dotted lines.

In order to assess the performance of Deep‐MSIM with fewer raw images, we use 274 frames instead of 1058 frames of raw images of the same microtubules structures in Figure 2 as the input (**Figure** **8**a). Although both of Deep‐MSIM and conventional MSIM have “good enough” performance for using 1058 frames of raw images, they lead to distinguishing outputs with 274 frames.

First, the reconstructed super‐resolution image in Figure 8c is compared to the result of conventional MSIM method in Figure 8b. Image quality improvement after Deep‐MSIM reconstruction is pronounced, where microtubule structures are produced with greater clarity, and regions highlighted in cyan solid and dotted squares are examples. By contrast, there are obvious discontinuities and unstructured regions in conventional MSIM reconstruction result, due to the lack of information. Figure 8e,g depicts the intensity profiles along the white lines. It can be seen that Deep‐MSIM is capable of detecting microtubule structures with fewer raw image, while conventional MSIM method with fewer raw data fails to resolve two close microtubules.

Next, by using the reconstruction result of 1058 frames of raw data with conventional MSIM method in Figure 2b as the ground truth, we quantify the performance of the reconstructed results in Figure 8b,c. These results are listed in **Table** **1**, where generally larger PSNR and SSIM and smaller NRMSE of the image indicate that the conventional MSIM images are inferior to our inference images, and Deep‐MSIM achieves an acceptable super‐resolution image with fourfold reduction in number of raw images and a much shorter time of 11.60 s.

**Table 1:** Comparisons of performance with two methods using fewer frames of raw images

Raw images Methods PSNR [dB] NRMSE MSE SSIM Runtime [s]	
274 frames	Deep‐MSIM 25.437 0.053 188.920 0.82 11.6 Conventional MSIM 25.301 0.054 194.950 0.74 31.3

**Figure 9:**
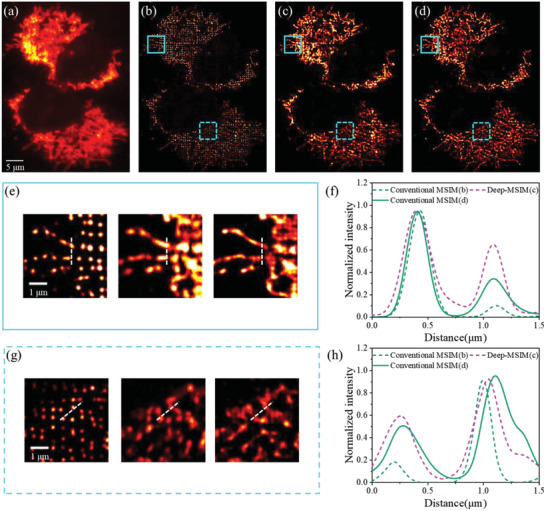
Comparison of conventional MSIM and Deep‐MSIM with fewer frames of raw experimental data. a) Wide‐field image summed from 121 frames of raw images. (b) and (c) are reconstructed images, respectively, using conventional MSIM and Deep‐MSIM with 121 frames of raw images. d) is reconstructed image using conventional MSIM with 442 frames of raw images. (e) and (g) are correspondingly magnified views of cyan solid and dotted squares. (f) and (h) are plots of intensity profiles along the white dotted lines.

Moreover, the trained model is applied to another different biological structure of mitochondria. By setting the step size as 2 in both horizontal and vertical direction, we capture 121 frames of raw images that are fourfold reduction in the number of raw images typically required for conventional MSIM reconstruction. The absence of raw data causes noticeable discontinuities in the reconstructed super‐resolution image (**Figure** **9**b) with conventional MSIM method, demonstrating its inability to clearly reveal complete structures of the mitochondria. As a deep‐learning‐based method, Deep‐MSIM obtains better reconstructions (Figure 9c) without losing much structural information, because it learns the feature representation in the training stage. We use the conventional MSIM reconstruction result (Figure 9d) from 442 frames of raw images as ground truth. The intensity profiles along the white dotted lines in Figure 9e,g are plotted in Figure 9f,h, where the lower intensity profile peaks manifests the failure of the conventional MSIM method in reconstructing the structures under insufficient amount of raw data condition, and Deep‐MSIM is able to super‐resolve closer and finer structures.

**Table** **2** also compares the measurements of PSNR, NRMSE, and SSIM for quantitative analysis. It is important to note that Deep‐MSIM exhibits a better performance with a shorter computation time. To reconstruct the super‐resolution images in Figure 9b,c, conventional MSIM method and Deep‐MSIM, respectively, spends 12.26 and 6.62 s in processing 121 frames of raw images, while conventional MSIM method needs a much longer time of 43.68 s to obtain Figure 9d by reconstructing 442 frames of raw images. The above results further affirm that our method successfully achieves speeding up MSIM by reducing the number of raw data required for reconstruction, and obtains super‐resolution image comparable to conventional MSIM method.

**Table 2:** Comparisons of performance with two methods using fewer frames of raw images

Raw images Methods PSNR [dB] NRMSE MSE SSIM Runtime [s]
121 frames Deep‐MSIM 27.483 0.042 117.565 0.82 6.62 Conventional MSIM 27.301 0.043 122.190 0.77 12.26

Although the two methods exhibit evidently different results in Figures 8 and 9, both the PSNR and NRMSE values of two methods are similar in Tables 1 and 2. This can be explained as follows. First, according to the formulations of calculating PSNR and NRMSE in Equation (10), the division by our large image sizes (509 × 448 for Figure 8 and 557 × 742 for Figure 9) lead to smaller numerical values and reduce their disparity. Second, the square root operation and normalization in the formulations further decrease the difference of PSNR and NRMSE between two methods. We add mean‐square‐error (MSE) values for comparison in Tables 1 and 2, and the proposed Deep‐MSIM obviously outperforms conventional MSIM in term of this metric. Third, because PSNR and NRMSE belong to the error‐sensitivity approach that is not very well matched to perceived visual quality,^[^ ^40^ ^]^ we also use SSIM to evaluate the performance of two methods when they achieve similar PSNR gain.

## 3 Discussion

In this paper, a deep‐learning‐based algorithm is developed for the reconstruction of super‐resolution images directly from raw MSIM images. Deep‐MSIM enables reduction in acquisition time compared to conventional MSIM method with acceptable spatial resolution, as reported in our Results.

We devise the network architecture based on the encoder‐decoder framework, and the two parts are combined in a way of dense connection that is introduced to strengthen the feature propagation, facilitate the learning process and obtain better performance. The strategy is beneficial for improving the image quality, which is demonstrated by comparing with several representative deep learning super‐resolution models. As a purely computational technique, Deep‐MSIM does not necessitate any changes to existing MSIM systems, and only requires simulated images for training. This is also one of the main reasons that deep learning is suitable for the application presented in this work, namely, it is straightforward to simulate a large number of training data with realistic noise models, which are often required in deep learning.

We use the same network model and different training data for static samples, live samples and fewer raw images, respectively. Our method can achieve super‐resolution image reconstruction, showing comparable or better performance in comparison with conventional reconstruction method of MSIM. Deep‐MSIM assists the investigation of dynamics of live thick samples at a greater depth by effectively rejecting the scattered and out‐of‐focus light. We also evaluate the reliability of the network output image, which is a valid concern for deep‐learning‐based algorithm. The error maps are beneficial for users identifying image regions where the reconstructed structures might not be accurate.

We use three‐fourths fewer frames of raw MSIM images to reconstruct super‐resolution image with favorable performance in terms of SSIM, PSNR, and NRMSE, improving the temporal resolution of MSIM in vivo imaging without increasing phototoxicity and photobleaching. Deep learning has brought about significant breakthroughs in single‐image super‐resolution (SISR) of realistic photographs, unfortunately, the demand for greater accuracy and higher fidelity of the nanoscale structures poses challenges for the application of deep‐learning‐based SISR to microscopy images. The limitation of the numbers of required raw MSIM images depends critically on the acceptability of the reconstructed results. More precise mappings from much fewer frames of raw images to super‐resolution output can be learnt by improving the network architecture, the quality of the training data and the design of the loss function on one hand; on the other hand, it is necessary to quantify the confidence in the inferred results, for example, what extent the information conveyed are reliable, and which areas of the image are problematic. Accordingly, we can make better decisions on using fewer frames of raw images for fast speed and short acquisition time, or using more frames for accurate structures.

All these results allow the proposed Deep‐MSIM to be a prime candidate for multifocal structured illumination microscopy, especially with the increasing demand for accurately and fast in vivo imaging of thick samples at an improved depth. It affords algorithmic insights to how the deep learning algorithm is applied to MSIM image reconstruction and reduces the time.

## 4 Experimental Section

### The Two‐Photon MSIM System

It mainly comprises two‐photon excitation and visible fluorescence detection, as illustrated in Figure S5, Supporting Information. The intensity of the femtosecond laser source (either a 920 nm (Spark Lasers, ALCOR 920) or a 1036 nm (YSL, Femto YLTM)) was controlled by a 1/2 λ wave plate (Thorlabs, AHWP05M‐980) and a polarizing beam splitter (PBS) cube (Thorlabs, CCM1‐ PBS252). The laser beam was then expanded by lenses (L1 and L2) and reflected to a high‐speed spatial light modulator (SLM, Meadowlark, HSP1920‐1152) at ≈10°. Another 1/2 λ wave plate was used to facilitate the desired polarization of the SLM. L3 and L4 create magnified phase patterns of the SLM at the rear pupil plane of the objective (Nikon, CFI Apo LWD 25X, 1.1 NA, and 2 mm WD). The fluorescence beam was focused by L5 and then captured by an EMCCD (Andor, Life 888, 1024 × 1024 pixels, 13 × 13 µm^2^).

### Sample Collection and Preparation

Zebrafish of Tg (Xla.Eef1a1:mlsEGFP) are purchased from China Zebrafish Resource Center as eggs and raised in E3 solution (containing 0.003% *N*‐phenylthiourea, Sigma) to inhibit pigmentation after 20 hpf. Before experiments, zebrafishes were anesthetized with 600 µm Tricaine (Sigma, Cat. # E10521), and then mounted for imaging in 1% low‐melting‐point agarose (Biosharp, Cat. # BS144). The experiments were reviewed and approved by the Experimental Animal Ethics Committee of Shenzhen University (A202200616).

### Architecture

Inspired by U‐net and MultiResUNet,^[^ ^43^ ^]^ the network architecture based on the encoder‐decoder framework was constructed, as shown in Figure S6, Supporting Information. The encoder and decoder in the network were connected with a two‐layer convolutional block and dense connections. U‐net and its variants were proposed to learn to suppress irrelevant regions while highlighting salient structures of varying shapes, yielding improved prediction performance across diverse datasets.^[^ ^36^ ^]^

The encoder network extracts spatial feature information from the input image and consists of three downsampling blocks. Let *d*_*i*,in_ and *d*_*i*,out_ be the input and output of the *i*thdownsampling block, respectively, and *d*_1,in_ be the low‐resolution input image. Each downsampling block includes two convolutional blocks, within which it performs

(1) $$\begin{array}{ccc} d_{i,\mathrm{out}} & = & \mathrm{ReLU}\left(\mathrm{BN}\left[\mathrm{Conv}\left(\mathrm{ReLU}\left(\mathrm{BN}\left[\mathrm{Conv}\left(d_{i,\mathrm{in}}\right)\right]\right)\right)\right]\right)\\ i & = & 1,2,3,4 \end{array}$$

in which Conv() represents the convolution operation with a stride of 1 and padding of 1. Convolutional filters of size 3×3 are first used to generate feature maps, followed by batch normalization (BN[]) to alleviate the internal covariate shift and speed up training^[^ ^44^ ^]^ and then the element‐wise rectified linear unit activation function ReLU() are added for nonlinearity.^[^ ^45^ ^]^ Mathematically, ReLU( · ) = max ( · , 0). The same kernel size 3×3 as that used in U‐net for better model accuracy and efficiency was selected. The width of each block denoted the number of filters in the corresponding convolutional layer, which is also the number of features in the representation. A max pooling layer MaxPool[] with stride 2 is used after the downsampling blocks to reduce the spatial dimension of the feature representation and help control overfitting.^[^ ^16^ ^]^ Max pooling performs down‐sampling operation by taking the maximal element in the corresponding 2×2 region and discarding redundant information.

The decoder network upsamples the feature maps to predict the super‐resolution image, and symmetrically comprises three upsampling blocks. Each upsampling block is also composed of two convolutional blocks, and its output can be derived as

(2) $$\begin{array}{ccc} u_{i,\mathrm{out}} & = & \mathrm{ReLU}\left(\;\mathrm{BN}\left[\;\mathrm{Conv}\left(\;\mathrm{ReLU}\left(\mathrm{BN}\left[\mathrm{Conv}\left(\mathrm{Concat}\left\{\mathrm{DC}\left(d_{4-i,\mathrm{out}}\right),\mathrm{UpS}\left(u_{i,\mathrm{in}}\right)\right\}\right)\right]\right)\right)\right]\right)\\ i & = & 1,2,3 \end{array}$$

where *u*_i,out_ and *u*_i,in_ are the output and input of the *i*th upsampling block, respectively, and *u*_1,in_ = *d*_4,out_. To avoid the checkerboard artifacts caused by regular transposed convolution operation,^[^ ^46^ ^]^ upsampling UpS() was separated out to a higher resolution from convolution to compute features. A nearest neighbor interpolation was first used to achieve spatial upsampling by a factor of two, and the ensuing 1×1 convolution layer reduces the feature channels by half. In addition, a dropout layer between successive upsampling blocks was inserted to alleviate overfitting, and the dropout probability was set as *p* = 0.5.

In the classical U‐net architecture, there was a concatenation operation Concat{, } between the corresponding levels of encoder and decoder as a skip connection, resulting in a possible semantic gap between the two sets of features being merged.^[^ ^43^ ^]^ Hence, instead of straight concatenation, it was proposed to combine the encoder feature maps with the decoder feature in a dense‐connection (DC) manner motivated by.^[^ ^47^ ^]^ There are three dense connections with different configurations of {*L* = 0}, {*L* = 3, *k* = 32}, and {*L* = 5, *k* = 8}, where *L* and *k* are the layer number and the growth rate of the network, respectively. The growth rate refers to the number of channels of each layer. Take the dense connection 3 illustrated in Figure S7a (Supporting Information) as an example, the input is first fed into a 3×3 convolutional layer. The bottleneck layer *H_l_* () is devised as BN‐ReLU‐Conv(1×1)‐BN‐ReLU‐Conv(3×3) to reduce its input channel numbers and improve computational efficiency. At the end of the dense connection, a transition layer consisting of BN‐ReLU‐Conv(1×1)‐BN‐ReLU is performed to change the number of channels back to the same as that of the input. Each layer has access to all preceding layers and passes on its own output to all subsequent layers, and the feature maps learned by different layers are concatenated to ensure maximum information flow in the network.^[^ ^47^ ^]^ As a result, the feature map *X_l_* produced in the *l*th layer is given by

(3) $$X_{l}=H_{l}\left(\mathrm{Concat}\left\{X_{0},X_{1},\text{…}X_{l-1}\right\}\right)$$

These dense connections are expected to strengthen feature propagation and reduce the semantic gap. Furthermore, they are also introduced to make the learning easier and obtain better performance.^[^ ^47^ ^]^

Another main part of the network architecture is the output block, and its detail is depicted in Figure S7b, Supporting Information. According to the conventional MSIM reconstruction methods, a scaling factor of 2 is chosen in their “local scaling” step, because they shift the light collected by each pixel toward the closest illumination focus by half the distance that separates the pixel from the focus.^[^ ^8^, ^10^ ^]^ This means that if the input is a raw MSIM image of *W×H* pixels, then the output is a super‐resolution image of *2W×2H* pixels. For the proposed Deep‐MSIM, the output should have the same pixels as that of input image, yet the output block was devised to implement the rescaling and also obtain a super‐resolution image of *2W×2H* pixels. A nearest neighbor interpolation was first used to achieve the double spatial size, and then two convolutional blocks with different kernel sizes were followed to map the 32 channels into 1 channel that corresponds to a monochrome grayscale high‐resolution image. Pseudocolor was applied for better visualization.

### Loss Function

The loss function as a combination of mean absolute error (MAE) and the multiscale structural similarity index (MS‐SSIM) was designed.^[^ ^48^ ^]^ The former measures the *l*_1_ norm of the difference between the network's prediction *Y* and the ground‐truth image $\bar{Y}$, while the latter enhances the perceptual quality of the output and preserves the contrast in high‐frequency regions.^[^ ^49^ ^]^ This leads to the following loss function

(4) $$L=\alpha \left(1-\mathrm{MS}-\mathrm{SSIM}\left(Y,\bar{Y}\right)\right)+\left(1-\alpha \right)G\cdot {\left\|Y-\bar{Y}\right\|}_{1}$$

in which *α* is parameter for the tradeoff between corresponding terms, and α=0.75 was selected empirically to achieve the best performance in the experiments. As in ref. [49], a point‐wise multiplication between *l*_1_ loss and a Gaussian kernel *G* of size 9×9 with standard deviation of 1.5 was also added. Given *M* scales of images generated by down‐sampling,^[^ ^48^ ^]^ MS‐SSIM is defined as

(5) $$\begin{array}{ccc} \mathrm{MS}-\mathrm{SSIM}\left(Y,\bar{Y}\right) & = & {\left[l_{M}\left(Y,\bar{Y}\right)\right]}^{\beta_{M}}\cdot \prod_{j=1}^{M}{\left[{\mathrm{cs}}_{j}\left(Y,\bar{Y}\right)\right]}^{\beta_{j}}\\ & = & {\left[\frac{2\mu_{Y}\mu_{\bar{Y}}+C_{1}}{\mu_{Y}^{2}+\mu_{\bar{Y}}^{2}+C_{1}}\right]}^{\beta_{M}}\cdot \prod_{j=1}^{M}{\left[\frac{2\sigma_{Y\bar{Y}}+C_{2}}{\sigma_{Y}^{2}+\sigma_{\bar{Y}}^{2}+C_{2}}\right]}^{\beta_{j}} \end{array}$$

where $l_{j}\left(Y,\bar{Y}\right)$ and $cs_{j}\left(Y,\bar{Y}\right)$ are the measures of luminance, contrast, and structure corresponding to scale *j*. The exponents β_*M*_ and β_*j*_ are used to adjust the relative importance of different components, and they are typically set as [0.1, 0.3, 0.3, 0.2, 0.1]. *µ* and *σ* represent the mean and standard deviation of the images, respectively, and $\sigma_{\bar{Y}Y}$ is the covariance of $\bar{Y}$ and *Y*. To prevent the denominator from becoming zero, both *C*_1_ and *C*_2_ are very small constants.

### Training

The network was trained solely on simulated data. Although the degradation process in simulation hardly be as complicated and accurate as that in real application, it was quite convenient to produce sufficiently large datasets for optimization. Especially for MSIM, its physical principle of imaging process has been well understood and it is straightforward to simulate raw data. A thin 2D “spoke‐like” sample (Figure 1b) was designed whose fluorescence density is given by

(6) $$\rho \left(r,\theta \right)\propto \left[1+\cos\left(48\theta \right)\right]$$

in which (*r*, θ) are the polar coordinates in the sample plane.^[^ ^15^ ^]^ The width of the spokes can be changed by substituting random values for 48. This radial sample is appropriate for studying the performance of imaging techniques.^[^ ^50^ ^]^

The training data pairs were divided into raw image and corresponding super‐resolution image. According to the physical principle of image process and the formulations in the previous work,^[^ ^14^, ^15^ ^]^ the multispot illumination patterns *p_i_* in MSIM can be described as

(7) $$p_{i}\left(u\right)=\sum_{j=1}^{n}\delta \left(r-b_{\mathrm{ij}}\right)⊗h_{\mathrm{ex}}\left(u\right),i=1,2,\cdot \cdot \cdot ,m$$

where δ() is the Dirac delta function and *h*_ex_ is the excitation PSF. *u* denotes the illumination field (on the specimen plane). *r* is the pattern field. *b*_ij_ represents the spatial positions of the *j*th single illumination spot (a total of *n* spots in each frame of illumination pattern) in the *i*th shifted illumination patterns (a total of *m* frames of illumination pattern), and ⊗ is convolution operator. By exciting the sample *s* with the multispot illumination patterns *p_i_*, first 256×256 pixels were generated (pixel resolution 130 nm) of raw multispot image as

(8) $$I_{i}\left(x\right)=\int p_{i}\left(u\right)s\left(u\right)h_{\mathrm{em}}\left(x-u\right)du$$

in which *h*_em_ is the emission PSF. *x* denotes the positions on the image plane. These simulated images are then corrupted by Gaussian noise and Poisson noise after a reasonable estimation of the experimental data to make the simulation more realistic.

Next, traditional MSIM reconstruction algorithm was applied^[^ ^8^, ^10^ ^]^ to these raw images to obtain the corresponding super‐resolution images as ground truth. The processing steps can be briefly summarized as follows. 1) Pinholing: The raw image is first denoised for more accurate lattice detection that determines the positions of the illumination spots by finding the local maxima. Then a 2D Gussian digital pinhole mask is applied to subimage that is centered around each detected illumination spot to reject out‐of‐focus light. 2) Local scaling: This step improves the resolution by $\sqrt{2}$ resampling the subimages. 3) Summing the processed images. 4) Deconvolution is employed to recover the full 2× resolution enhancement. The code of steps (1)–(3) is written with MATLAB, and the deconvolution is implemented with DeconvolutionLab2 plugin in ImageJ.

Eventually 5120 pairs were prepared of low‐resolution images and high‐resolution images, of which 70% were randomly selected as the training set and the others were the validation data. All the data were normalized by dividing by the maximum intensity value of the dataset, which facilitated the network training and robustness enhancement.

The model was initialized by default and optimized using the adaptive moment estimation (Adam) optimizer,^[^ ^51^ ^]^ with a starting learning rate of 0.001. This framework was implemented with Pytorch^[^ ^52^ ^]^ framework version 1.7.1 and Python version 3.6.13 in the Microsoft Windows 10 operating system. In order to save the memory and accelerate the training speed, the automatic mixed precision within Pytorch was employed. The training and the inference of Deep‐MSIM were performed on a consumer‐grade laptop (Alienware‐51r, Dell) equipped with a GeForce RTX2080 graphics card (NVIDIA), while the conventional method was performed on MATLAB 2020b with a Core i9‐9900K CPU @ 3.6 GHz (Intel) and accelerated with parallel computing. The network was trained for 50 epochs on batch size of 8 due to the limitation of the graphics memory, reaching the minimum validation loss value, which was sufficient for different images in the experiments. It took ≈5 h to train the network with simulated training data. Proper settings were supposed to further promote the speed of the proposed method. Note that, the network needs to be trained only one time under the same imaging conditions, and the resulting model and the optimized weights are appropriate to reconstruct different biological structures and samples.

### Ablation Study

Ablation studies were conducted to investigate the individual contributions of each improved component in the network, including the batch normalization, the dense connections, the upsampling in the decoder, the output block, the dropout layer, and α=0.75 in the loss function. Specifically, following experiments were performed respectively: 1) the dense connection with regular concatenation and residual block, respectively, was replaced. 2) The output block was deleted. 3) Regular transposed convolution was used instead of the upsampling in the decoder. 4) All the BN and the dropout layer in the model, respectively, was deleted. 5) The value of *α* to 0.5 and 0.25, respectively, was changed. The ablation studies are not only tested on the sparse microtubules in Figure 2 and the mitochondria in Figure 4, but also on a new structure of densely distributed microtubules, and their corresponding results are presented in Figures S8, S9, and S10 (Supporting Information), respectively. With conventional MSIM reconstruction as ground truth, the results in the metrics of SSIM, PSNR, NRMSE, and MSE (Tables S3, S4, and S5, Supporting Information) were also carefully quantified. It can be demonstrated from the figures and tables that inclusions of such improved components in the network were able to boost the reconstruction performance. Deep‐MSIM acquires the best PSNR and NRMSE for sparse microtubules, the highest SSIM for dense microtubules, as well as the top two results for mitochondria. The architecture of Deep‐MSIM enables it to achieve superior image quality for different biological structures.

### Quantitative Evaluation of Image

The RSE and RSP of the NanoJ‐SQUIRREL plugin are formulated as

(9) $$\begin{array}{ccc} \mathrm{RSE} & = & \sqrt{\frac{\sum_{x,y}{\left(I_{D}\left(x,y\right)-I_{\mathrm{RS}}\left(x,y\right)\right)}^{2}}{N}}\\ \mathrm{RSP} & = & \frac{\sum_{x,y}\left(I_{D}\left(x,y\right)-{\bar{I}}_{D}\right)\left(I_{\mathrm{RS}}\left(x,y\right)-{\bar{I}}_{\mathrm{RS}}\right)}{\sqrt{\sum_{x,y}\left(I_{D}\left(x,y\right)-{\bar{I}}_{D}\right)}\sqrt{\sum_{x,y}\left(I_{\mathrm{RS}}\left(x,y\right)-{\bar{I}}_{\mathrm{RS}}\right)}} \end{array}$$

where ${\bar{I}}_{D}$represents the average value of the reference diffraction‐limited image *I*_D_, and ${\bar{I}}_{\mathrm{RS}}$ denotes the average value of the resolution‐scaled image *I*_RS_ that is created by applying the resolution scaling function to the super‐resolution image. Here *N* represents the total number of pixels.

In this work, the three metrics of PSNR, NRMSE, and SSIM were examined to assess the overall performance of the models. They are calculated, respectively, by

(10) $$\begin{array}{ccc} \mathrm{PSNR}\left(Y,\bar{Y}\right) & = & -20\times \log\left(\sqrt{\sum_{i=1}^{w}{\sum_{j=1}^{h}\left(\bar{Y}\left(i,j\right)-Y\left(i,j\right)\right)}^{2}/\left(w\times h\right)}\right)\\ \mathrm{NRMSE}\left(Y,\bar{Y}\right) & = & \in \sqrt{\frac{1}{w\times h}\sum_{i=1}^{w}{\sum_{j=1}^{h}\left(\bar{Y}\left(i,j\right)-Y\left(i,j\right)\right)}^{2}/\left(\max\left(Y\right)-\min\left(Y\right)\right)}\\ \mathrm{and}\mathrm{SSIM}\left(Y,\bar{Y}\right) & = & \left(\frac{2\mu_{Y}\mu_{\bar{Y}}+c_{1}}{\mu_{Y}^{2}+\mu_{\bar{Y}}^{2}+c_{1}}\right)\cdot \left(\frac{2\sigma_{Y\bar{Y}}+c_{2}}{\sigma_{Y}^{2}+\sigma_{\bar{Y}}^{2}+c_{2}}\right) \end{array}$$

in which the width and height of the images are denoted by *w* and *h*, respectively. *µ* and *σ* represent the mean and standard deviation of the images, respectively, and $\sigma_{Y\bar{Y}}$ is the covariance of the network output *Y* and the ground‐truth image $\bar{Y}$. Both *c*_1_ and *c*_2_ are very small constants to stabilize the division. The code for the calculating PSNR and NRMSE is written with Python, while the SSIM is estimated using SSIM index plugin in ImageJ with the default parameter settings. All of them are done with 8‐bit images.

### Statistical Analysis

The image size of the cropped patches in Figures 1 and 3, 4, 5, 6 are 512 × 512 pixels, 639 × 635 pixels, 635 × 807 pixels, 620 × 631 pixels, and 796 × 796 pixels, respectively. The FWHM of the intensity file in Figures 4g and 5g are performed by Origin 2021.

## Conflict of Interest

The authors declare no conflict of interest.

## Supporting information

Supporting Information

Supplemental Video 1

Supplemental Video 2

## Data Availability

The data that support the findings of this study are available from the corresponding author upon reasonable request.
