# Supplementary material for: Deep‐MSIM: Fast Image Reconstruction with Deep Learning in Multifocal Structured Illumination Microscopy
Source: Adv Sci (Weinh). 2023 Jul 9;10(27):2300947. doi: 10.1002/advs.202300947 (PMC10520669; doi:10.1002/advs.202300947)
Supplement: Supplementary file 1 — Supporting Information [file ADVS-10-2300947-s002.pdf]

ADVANCED SCIENCE
Open Access

Supporting Information

for *Adv. Sci.*, DOI 10.1002/advs.202300947

Deep-MSIM: Fast Image Reconstruction with Deep Learning in Multifocal Structured Illumination Microscopy

Jianhui Liao, Chenshuang Zhang, Xiangcong Xu, Liangliang Zhou, Bin Yu, Danying Lin, Jia Li and Junle Qu**

Supporting Information

Deep-MSIM: Fast Image Reconstruction with Deep Learning in Multifocal Structured Illumination Microscopy

Jianhui Liao[#], Chenshuang Zhang[#], Xiangcong Xu, Liangliang Zhou, Bin Yu, Danying Lin, Jia Li[*], and Junle Qu[*]
State Key Laboratory of Radio Frequency Heterogeneous Integration, Key Laboratory of Optoelectronic Devices and Systems of Ministry of Education and Guangdong Province, College of Physics and Optoelectronic Engineering, Shenzhen University, Shenzhen 518060, China
[#]These authors contributed equally.
[*]Corresponding authors: jli@szu.edu.cn; jlqu@szu.edu.cn

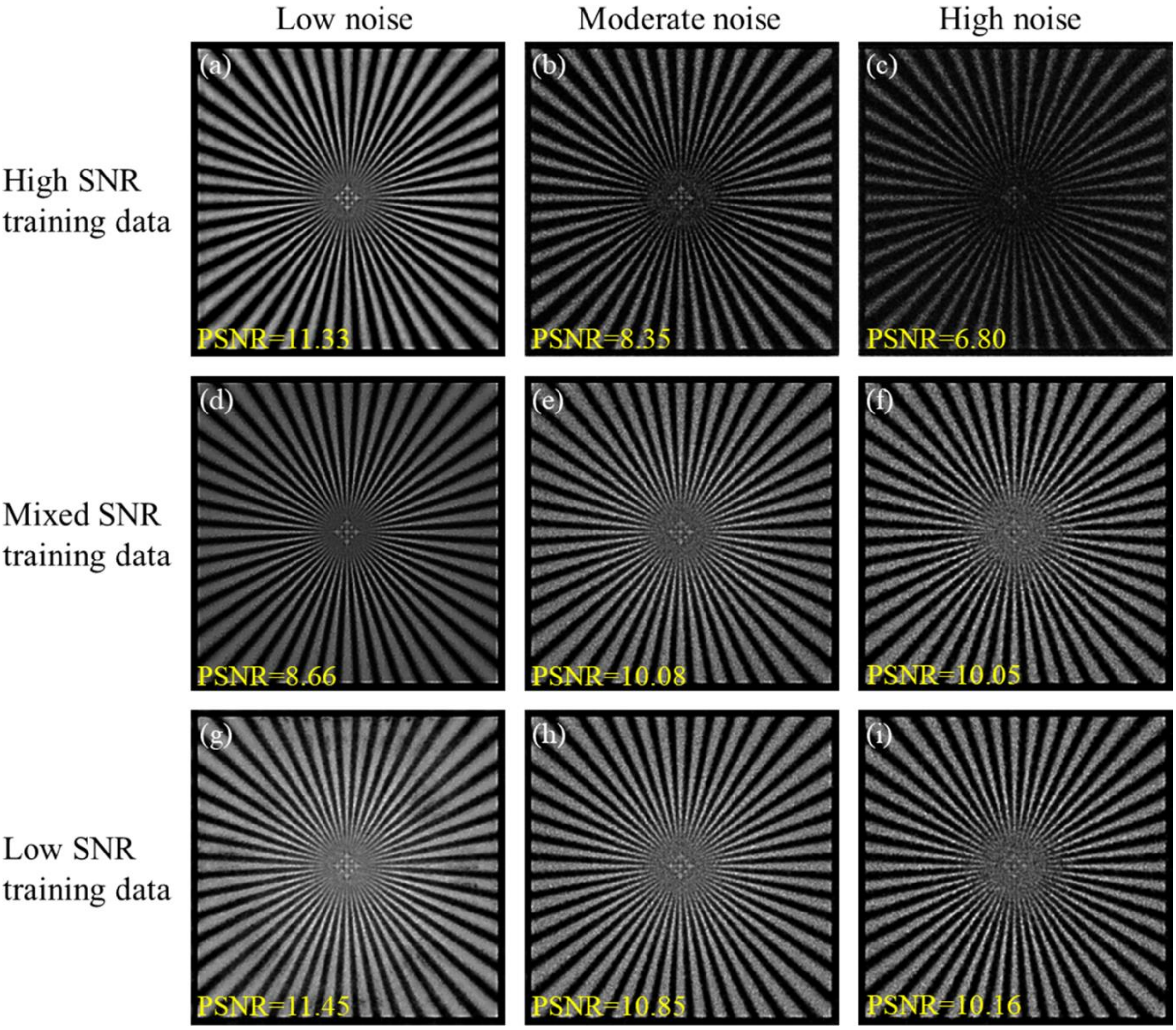

Figure S1. Comparison of Deep-MSIM trained with different SNR data on simulated image with different noise levels. a)-c) are reconstructed images from raw images respectively with low noise, moderate noise and high noise using Deep-MSIM trained with high SNR data. d)-f) are reconstructed images from raw images respectively with low noise, moderate noise and high noise using Deep-MSIM trained with mixed SNR data. g)-i) are reconstructed images from raw images respectively with low noise, moderate noise and high noise using Deep-MSIM trained with low SNR data.

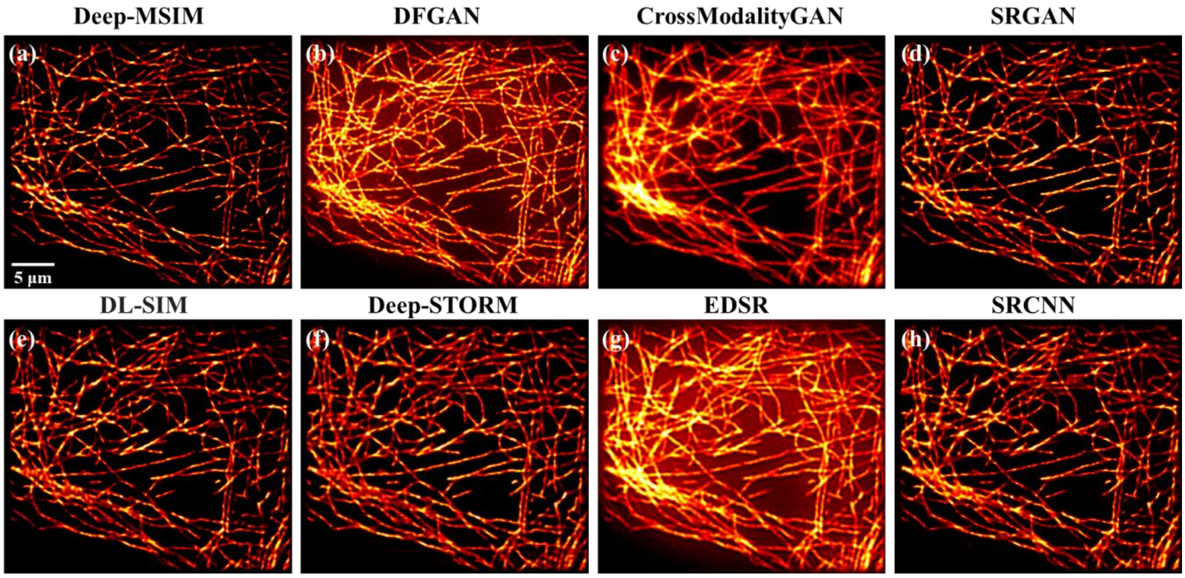

Figure S2. Comparison of different deep-learning-based super-resolution models on experimental data of sparse microtubules.

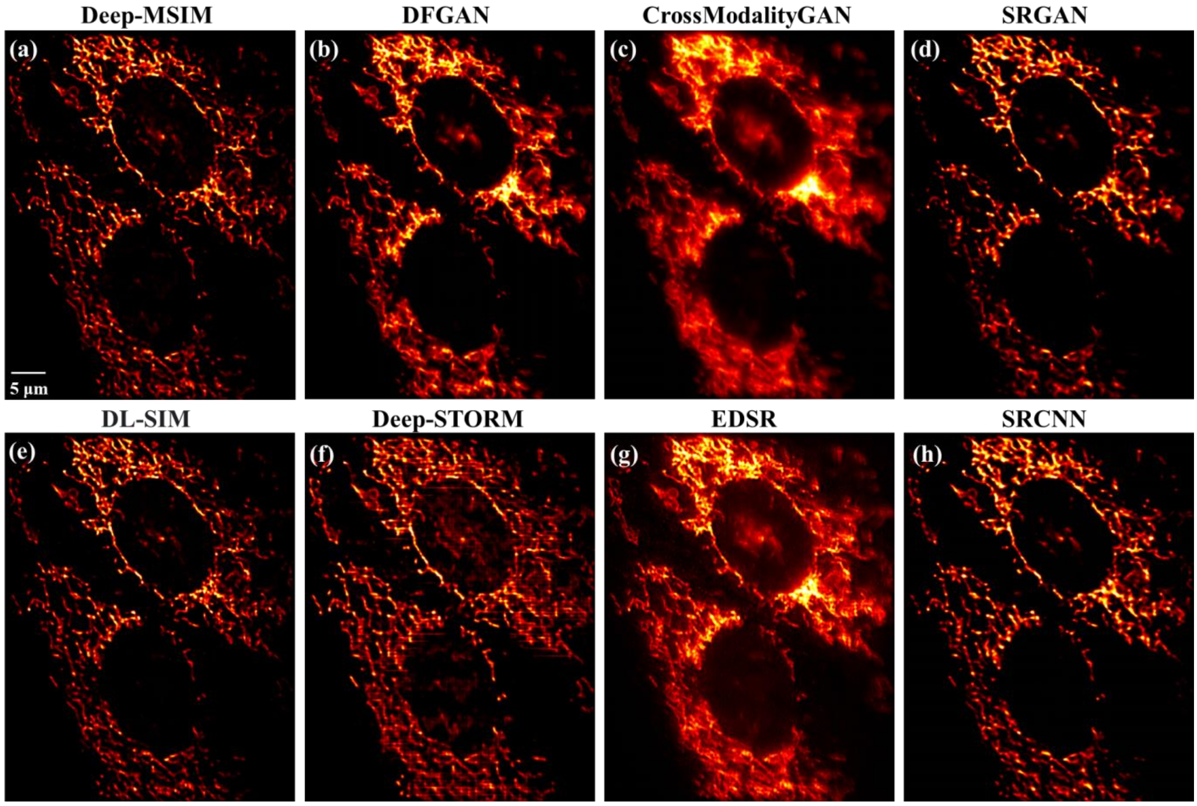

Figure S3. Comparison of different deep-learning-based super-resolution models on experimental data of mitochondria.

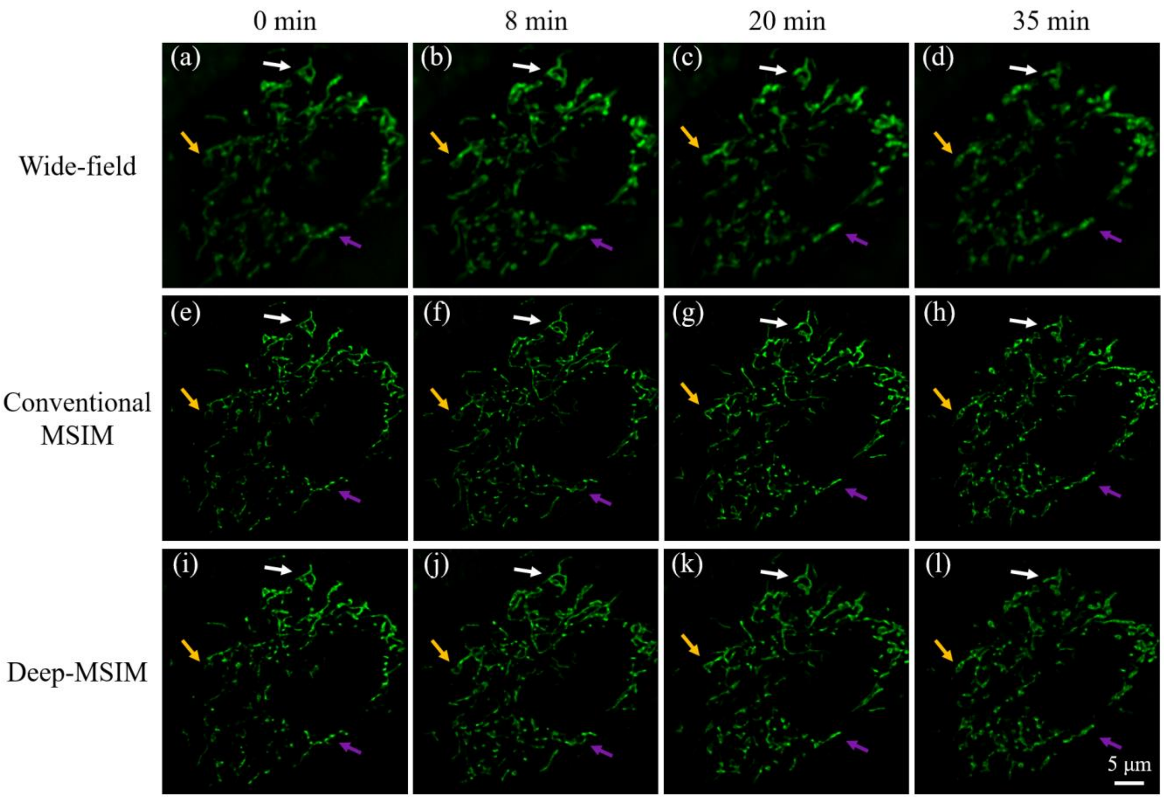

Figure S4. Comparison of conventional MSIM and Deep-MSIM on experimental data of live mitochondria. a)-d) are time-lapse wide-field image summed from 1600 frames of raw MSIM images. e)-h) are time-lapse reconstructed images using conventional MSIM. i)-l) are time-lapse reconstructed images using Deep-MSIM. The morphological changes are indicated by white, magenta, green and yellow arrows. Supplementary Video 2 shows the same field of view to further highlight the success of Deep-MSIM in revealing mitochondrial dynamics.

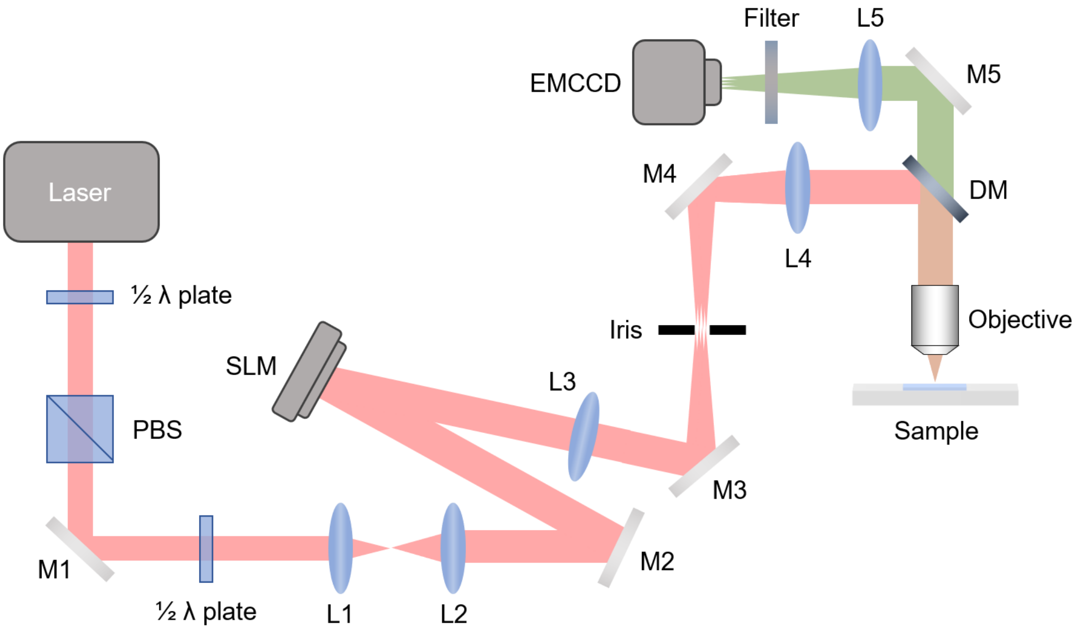

Figure S5. Schematic diagram of two-photon MSIM system. L1-L5: lenses. M1-M5: mirrors.

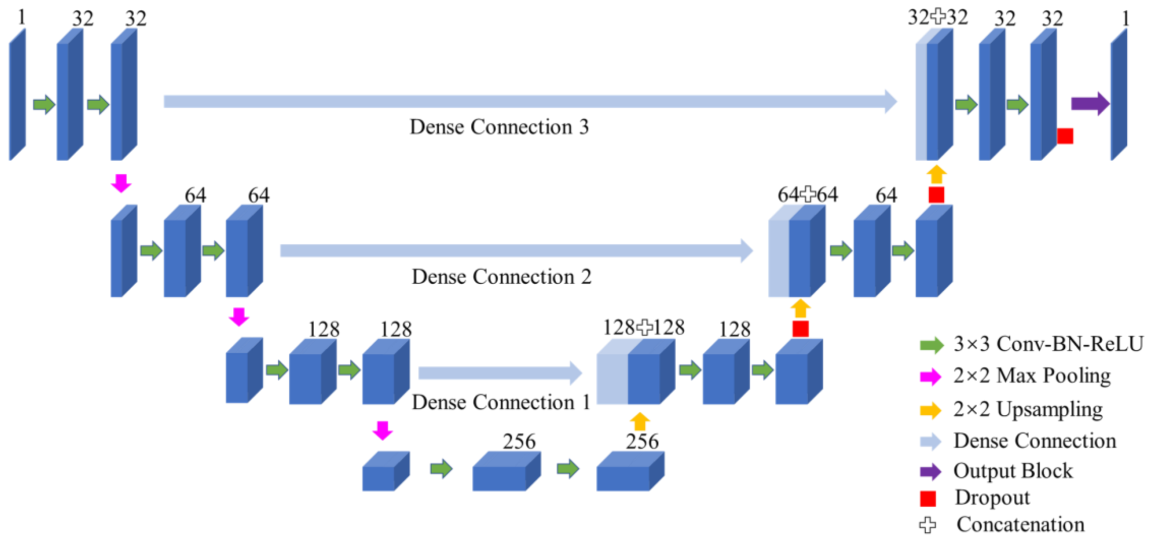

Figure S6. The network architecture of the proposed Deep-MSIM.

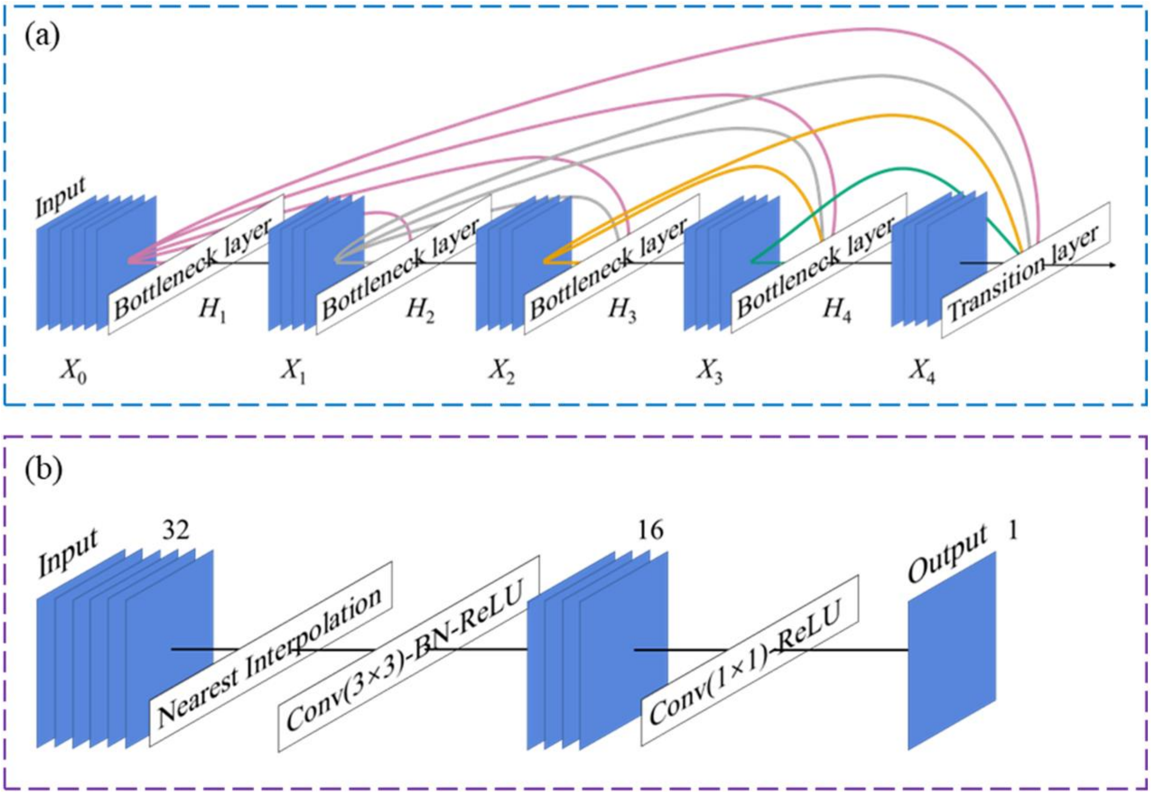

Figure S7. a) Dense connection 3: a 5-layer dense block with a growth rate of $k=8$. b) The output block.

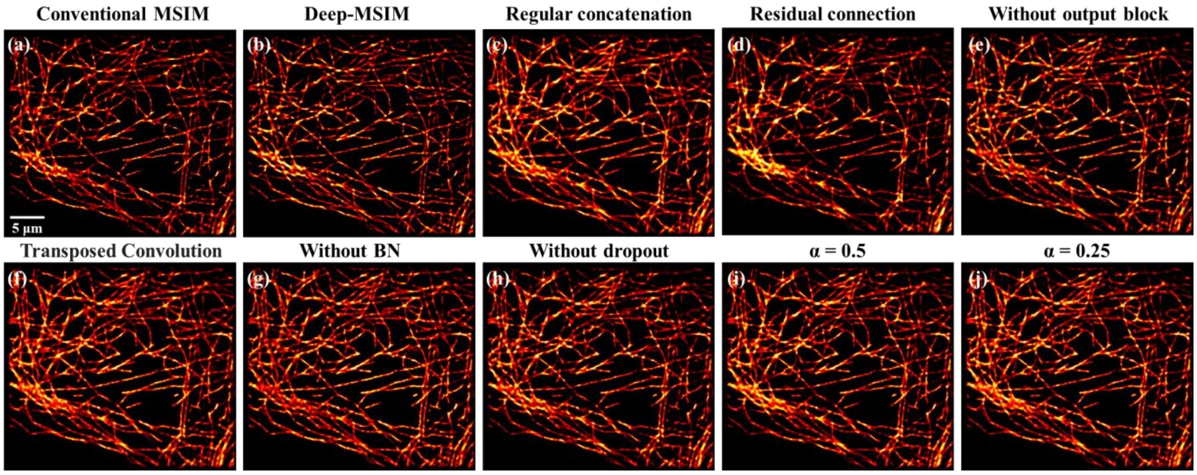

Figure S8. Ablation studies investigating the individual contributions of each improved components. a) to j) are reconstructed images of sparse microtubules respectively using conventional MSIM, Deep-MSIM, with regular concatenation, with residual connection, without output block, with transposed convolution, without batch normalization, without dropout, α=0.5 and α=0.25.

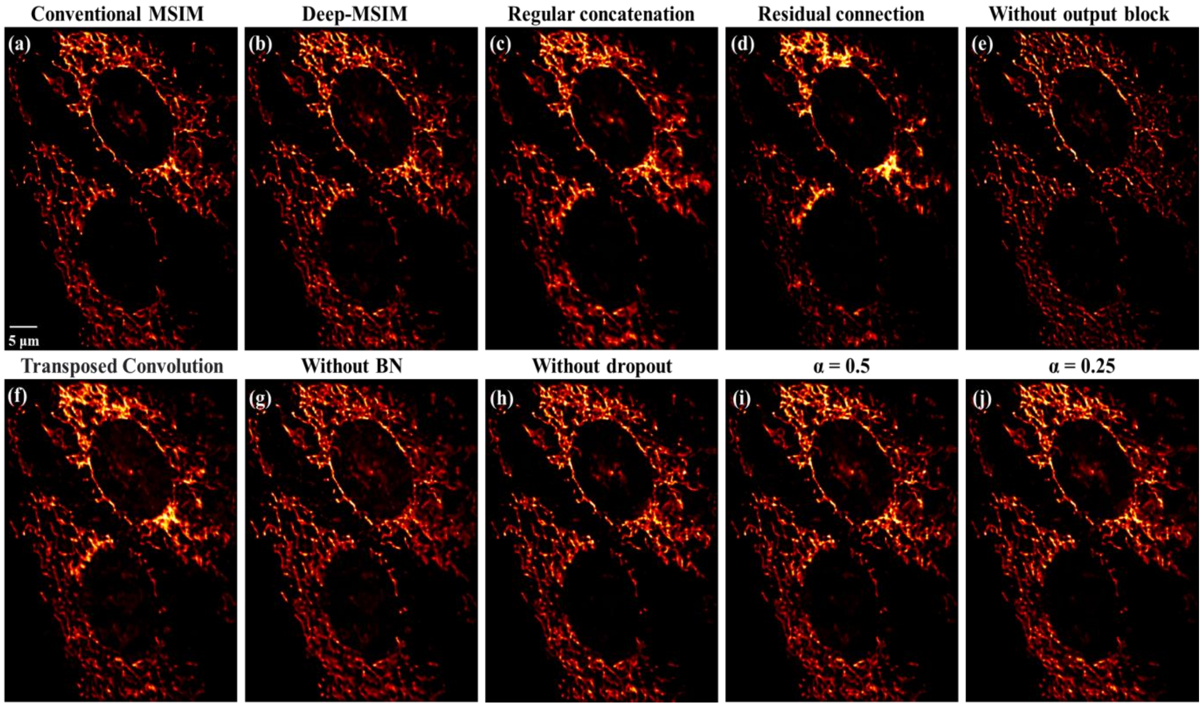

Figure S9. Ablation studies investigating the individual contributions of each improved components. a) to j) are reconstructed images of mitochondria respectively using conventional MSIM, Deep-MSIM, with regular concatenation, with residual connection, without output block, with transposed convolution, without batch normalization, without dropout, α=0.5 and α=0.25.

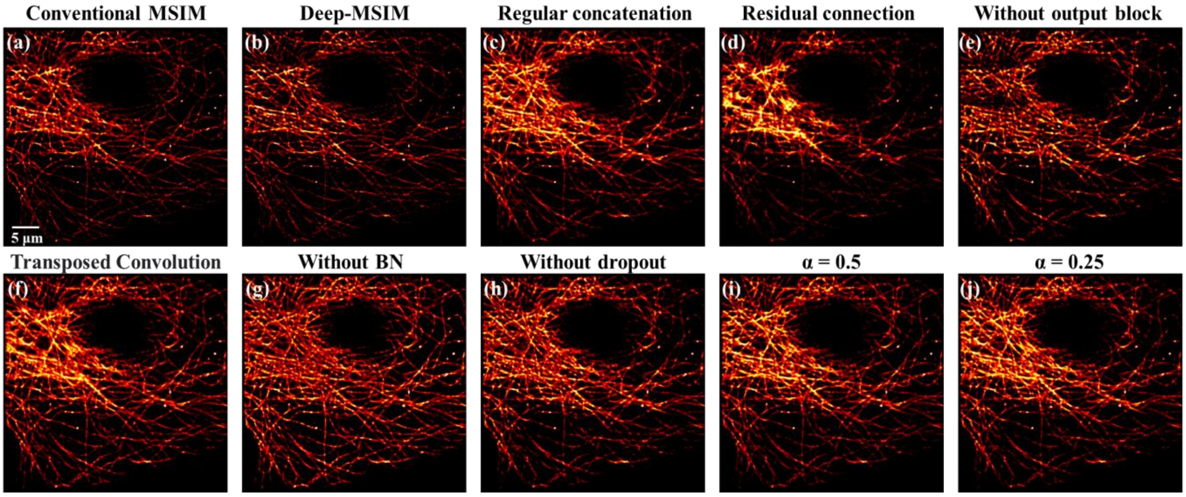

Figure S10. Ablation studies investigating the individual contributions of each improved components. a) to j) are reconstructed images of dense microtubules respectively using conventional MSIM, Deep-MSIM, with regular concatenation, with residual connection, without output block, with transposed convolution, without batch normalization, without dropout, α=0.5 and α=0.25.

Table S1. Quantitative results of SSIM, PSNR and NRMSE of all evaluated super-resolution models on experimental data of sparse microtubules. The best results are highlighted in bold.

Methods	SSIM	PSNR(dB)	NRMSE
Deep-MSIM	**0.753**	**25.065**	**0.055**
DFGAN	0.493	20.984	0.089
CrossModalityGAN	0.491	21.051	0.089
SRGAN	0.692	21.779	0.081
DL-SIM	0.743	23.911	0.063
Deep-STORM	0.595	20.925	0.089
EDSR	0.146	21.284	0.086
SRCNN	0.4	20.495	0.094

Table S2. Quantitative results of SSIM, PSNR and NRMSE of all evaluated super-resolution models on experimental data of mitochondria. The best results are highlighted in bold.

Methods	SSIM	PSNR(dB)	NRMSE
Deep-MSIM	**0.826**	**31.736**	**0.026**
DFGAN	0.601	23.942	0.064
CrossModalityGAN	0.578	23.835	0.064
SRGAN	0.808	25.735	0.052
DL-SIM	0.87	31.419	0.027
Deep-STORM	0.679	28.277	0.039
EDSR	0.1	20.969	0.089
SRCNN	0.568	25.642	0.052

Table S3. Comparisons of performance of reconstructed sparse microtubules for ablation studies investigating the individual contributions of each improved components. The best results are highlighted in bold.

Methods	SSIM	PSNR(dB)	NRMSE	MSE
Deep-MSIM	0.753	**25.065**	**0.055**	**202.567**
Regular concatenation	0.742	22.275	0.076	385.110
Residual connection	0.771	22.692	0.073	349.883
Without output block	0.756	23.854	0.064	267.723
Transposed convolution	0.731	24.220	0.061	246.070
Without BN	0.768	24.501	0.059	230.680
Without dropout	**0.791**	24.883	0.057	211.264
$\alpha = 0.5$	0.766	23.219	0.068	309.886
$\alpha = 0.25$	0.636	22.765	0.072	343.984

Table S4. Comparisons of performance of reconstructed mitochondria for ablation studies investigating the individual contributions of each improved components. The best results are highlighted in bold.

Methods	SSIM	PSNR(dB)	NRMSE	MSE
Deep-MSIM	0.826	31.736	0.026	43.604
Regular concatenation	0.836	29.077	0.035	80.416
Residual connection	0.824	27.804	0.040	107.804
Without output block	0.868	28.442	0.038	93.079
Transposed convolution	0.685	28.276	0.038	96.721
Without BN	0.760	30.461	0.030	58.474
Without dropout	**0.877**	**31.821**	**0.025**	**42.754**
$\alpha = 0.5$	0.781	29.286	0.034	76.646
$\alpha = 0.25$	0.813	29.554	0.033	72.054

Table S5. Comparisons of performance of reconstructed dense microtubules for ablation studies investigating the individual contributions of each improved components. The best results are highlighted in bold.

Methods	SSIM	PSNR(dB)	NRMSE	MSE
Deep-MSIM	**0.910**	35.370	0.017	18.881
Regular concatenation	0.688	30.465	0.030	58.426
Residual connection	0.743	23.757	0.064	273.791
Without output block	0.801	30.465	0.030	58.426
Transposed convolution	0.741	28.953	0.035	82.748
Without BN	0.810	33.806	0.022	31.950
Without dropout	0.800	30.530	0.030	57.558
$\alpha = 0.5$	0.739	27.975	0.040	103.657
$\alpha = 0.25$	0.889	**36.447**	**0.015**	**14.738**
